# White Matter Hyperintensities and Driving Behavior: Mitigating Effects of Hypertension Treatment

**DOI:** 10.1093/geroni/igaf122.376

**Published:** 2025-12-31

**Authors:** David Carr, Madhur Parihar, Jin-Moo Lee, Ganesh Babulal, Yasheng Chen, Chia-Ling Phuah

**Affiliations:** Washington University in St. Louis, St Louis, Missouri, United States; Barrow Neurological Institute, Phoenix, Arizona, United States; Washington University in St. Louis, St. Louis, Missouri, United States; Washington University School of Medicine in St. Louis, SAINT LOUIS, Missouri, United States; WashU Medicine, St. Louis, Missouri, United States; Barrow Neurological Institute, Phoenix, Arizona, United States

## Abstract

White matter hyperintensities (WMH) compromise cognitive reserve, potentially accelerating dementia onset. We investigated WMH’s impact on complex cognitive performance through longitudinal analysis of naturalistic driving behavior in older adults (OA). We examined 212 cognitively intact OA (aged >65 years, CDR=0) in the DRIVES Project cohort, analyzing 16 driving metrics using in-vehicle dataloggers, and quantifying WMH using deep learning. Linear mixed-effects models, adjusted for demographics, socioeconomic status, and composite vascular risk, assessed WMH influence on longitudinal changes in driving performance. Our study analyzed 74,275 weeks of driving data (2015-2024, average follow-up of 6.1 years). Higher WMH burden correlated with decreased trip frequency, fewer near-home trips, reduced unique destinations, and lower driving entropy over time (all p < 0.002). Posterior WMH lesions, particularly in parietal and occipital regions, were primarily linked to driving complexity decline (beta=-0.16, p = 0.009, beta =-0.15, p = 0.01, respectively). WMH impact intensified in participants developing cognitive impairment (n = 36), manifesting as increased hard braking and impact events. Anti-hypertensive medication users (n = 147) showed less decline in driving frequency and complexity (p = 0.03). WMH in OA significantly impacts driving behavior, leading to latent self-regulation and reduced driving complexity. Anti-hypertensive use may mitigate WMH effects on driving behavior change over time, despite posterior WMH suggesting a prominent role for AD in driving decline, warranting further study of blood pressure treatment. WMH shows potential as a biomarker for identifying individuals at higher risk of unsafe driving and early cessation, underscoring its value in screening and intervention for road safety among aging populations.

